# Supplementary material for: Comparative Analysis of Volatile Compounds of Gamma-Irradiated Mutants of Rose (Rosa hybrida)
Source: Plants (Basel). 2020 Sep 17;9(9):1221. doi: 10.3390/plants9091221 (PMC7569881; doi:10.3390/plants9091221)
Supplement: Supplementary file 1 [file plants-09-01221-s001.zip › supplementary/Supplementary figures.docx]

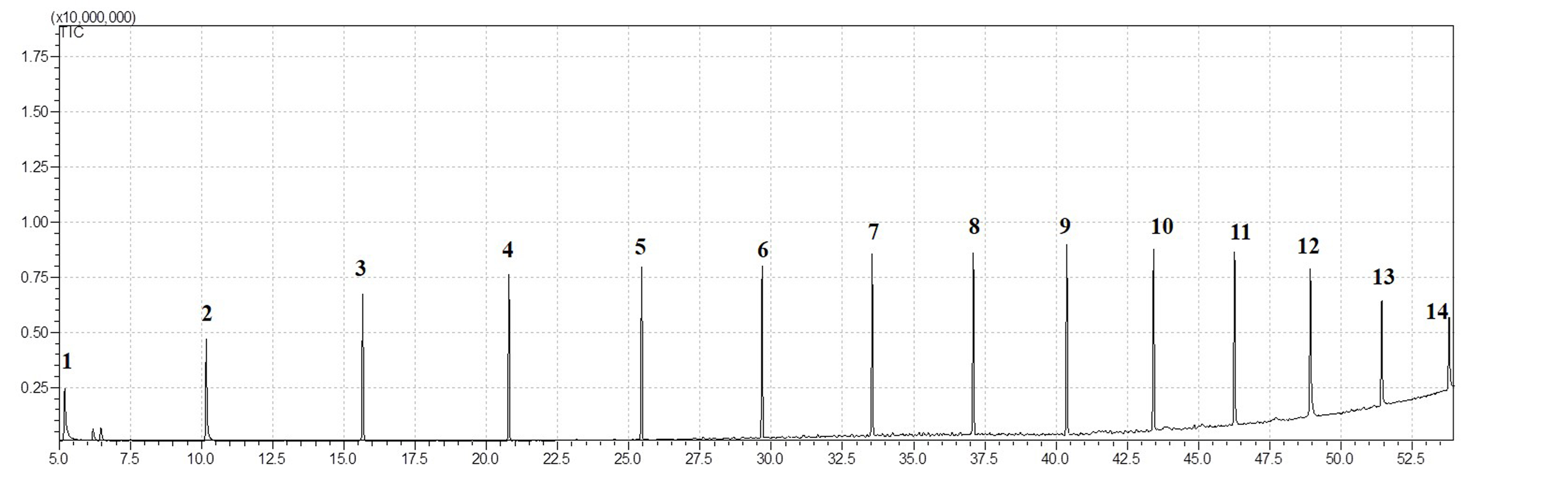


**Figure 1.** GC-MS chromatograms of the alkane standards. **1**: Decane, **2**: Dodecane, **3**: Tetradecane, **4**: Hexadecane, **5**: Octadecane, **6**: Eicosane, **7**: Docosane, **8**: Tetracosane, **9**: Hexacosane, **10**: Octacosane, **11**: Triacontane, **12**: Dotriacontane, **13**: Tetratriacontane,**14**: Hexatriacontane.

**
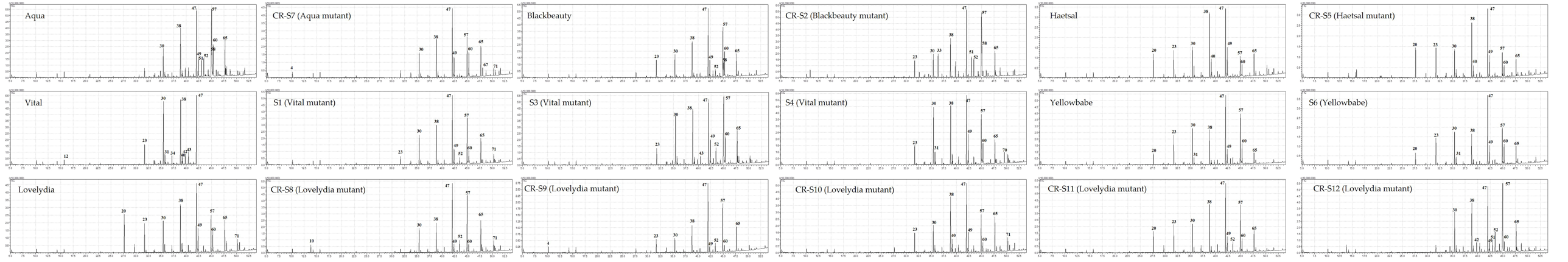
**

**Figure 2.** GC-MS chromatograms of the top 10 constituents identified in volatile compounds of the rose genotypes.
